# Gout Treatment and Clinical Considerations: The Role of Pegloticase, Colchicine, and Febuxostat

**DOI:** 10.7759/cureus.46649

**Published:** 2023-10-07

**Authors:** Michael J Quintana, Alika Z Shum, Michael S Folse, Prerana C Ramesh, Shahab Ahmadzadeh, Giustino Varrassi, Sahar Shekoohi, Alan D Kaye

**Affiliations:** 1 School of Medicine, Louisiana State University Health Sciences Center, Shreveport, USA; 2 Radiology, Louisiana State University Health Sciences Center, Shreveport, USA; 3 Anesthesiology, Louisiana State University Health Sciences Center, Shreveport, USA; 4 Pain Medicine, Paolo Procacci Foundation, Rome, ITA

**Keywords:** uric acid, gout, krystexxa (pegloticase), colcrys (colchicine), uloric (febuxostat)

## Abstract

Gout treatment has evolved rapidly in recent decades, and various drugs have been designed for acute and chronic management. Three medications used to treat gout include pegloticase, colchicine, and febuxostat. When prescribing these drugs, important factors to consider include pharmacokinetics, pharmacodynamics, population specifics, benefits, and contraindications. Pharmacokinetic considerations of each drug include absorption, distribution, metabolism, and elimination factors. Pharmacodynamics factors are assessed by their potential for toxicity and effects on serum uric acid levels. Additionally, the drug’s targeted population must be considered to avoid unwanted complications in certain pre-existing conditions such as cardiovascular disease or glucose-6-dehydrogenase (G6PD) deficiency. In this paper, we aim to provide insight into the gout medications, pegloticase, colchicine, and febuxostat. This review will include their pharmacokinetics, pharmacodynamics, population specifics, benefits, and contraindications.

## Introduction and background

In the United States, it is not uncommon for people to visit the hospital for acute gout, chronic (tophaceous) gout, and gout-related issues [1]. The pain associated with gout has been described by many as the worst pain they have experienced. Medical students across the country are taught to recognize gout based on the pain out of proportion it causes patients. Thus, this pain has an enormous impact on the quality of life of the affected population.

Gout is one of the earliest diseases to be documented and was first identified by the Egyptians as early as 2640 BC [2]. Some of the common names of gout throughout the years include "The Disease of Kings" and "The Unwalkable Disease" by Hippocrates, and eventually, the term "gout" was used by a Dominican monk named Randolphus of Bocking in the 1200s [2]. What would become the currently known condition of gout stemmed throughout history from an unexplainable pain in the first metatarsophalangeal (MTP) joint, also known as podagra. Gout is defined as inflammation of joints related to the precipitation of monosodium urate (MSU) crystals in the space between joints [3]. The creation of these MSU crystals comes from elevated urate concentration levels that are associated with hyperuricemia. Hyperuricemia can occur from numerous mechanisms, including genetic dispositions. Some main mechanisms of hyperuricemia are the overproduction of uric acid through enzyme defects, increased systemic nucleic acid related to high cell turnover, and increased purine uptake [3]. While this relationship is important, hyperuricemia alone is not diagnostic for gout.

There are numerous ways to narrow down a diagnosis of gout, but the gold standard for a definitive diagnosis is through a synovial fluid analysis. This technique works by aspirating synovial fluid from an inflamed joint and observing the fluid under polarized light using light microscopy. Under the microscope, rod or needle-shaped crystals with negative birefringence are seen with a vibrant yellow or blue color. This color relates to the crystals' position in the polarizing light, with yellow parallel and blue perpendicular. These characteristic colors and shapes are essential in differentiating gout from calcium pyrophosphate (CPP) crystals, also known as pseudogout [4]. Acute gout is diagnosed when observed neutrophils have intercellular MSU crystals. In contrast, a chronic gout diagnosis can occur by observing chalk-like subcutaneous nodules/deposits of MSU crystals (tophi) [3].

Acute and chronic (tophaceous) gout treatments have many similarities but differ in intensity. In both treatments, the primary goal is to control the pain and inflammatory response caused by the MSU crystal deposition. This is why non-steroidal anti-inflammatory drugs (NSAIDs), colchicine, or systemic glucocorticoids are the first-line treatment for gout [3]. Medications diverge from symptomatic relief for chronic gout to primarily acting prophylactically to prevent future attacks [3]. In the present investigation, therefore, the evaluation of current gout treatments is reviewed with a focus on febuxostat (brand name: Uloric), colchicine (brand name: Colcrys), and pegloticase (brand name: Krystexxa). The present investigation aims to consolidate the mechanism of action, benefits, contraindications, and targeted population for these three gout medications. Therefore, we hope to assist practitioners in making a more educated and fitting choice of treatment given a unique gout presentation.

## Review

Pharmacokinetics of gout drugs: Uloric, Colcrys, and Krystexxa

This section outlines the pharmacokinetic properties of the following gout drugs: Uloric, Colcrys, and Krystexxa.

Absorption

Absorption describes the way the body takes up a drug. The primary form of administration of Uloric is oral, taken once daily [5]. It has been noted that the average time to attain the maximum peak plasma concentration for Uloric is 0.5 and 1.5 hours under fasting conditions [5]. One study estimated the oral bioavailability to be about 75% and 85% based on the recovery of Uloric from feces and urine, respectively [5]. The absorption percentage is estimated to be about 49% [6]. Colcrys is primarily administered orally, once or twice daily, in adults and adolescents older than 16 [7]. Sites of absorption include the jejunum and ileum [8]. Colcrys has been noted to achieve a peak serum concentration of 2.5 ng/mL in a healthy adult in approximately one to two hours [9]. Regarding the absorption of Krystexxa, a 2012 study involving 23 patients who were administered 0.5-12 mg of Krystexxa found that the serum concentration increased proportionally to the administration dose [10].

Distribution 

Distribution describes where a drug goes following absorption. Uloric has a low volume of distribution (Vd) and primarily binds to albumin [11]. The plasma protein binding has been noted to be 99% and indicates the presence of Uloric in the circulation is high following administration [12,5]. The degree of plasma protein binding for Colcrys has been described previously as moderate [7]. The average level of binding reflects the large Vd associated with Colcrys [7]. A large Vd indicates that Colcrys is more likely to be found in tissue rather than circulation following administration. For Krystexxa, the same study by the FDA in 2012 found that factors affecting the Vd of Krystexxa included the presence of anti-pegloticase antibodies and body surface area [10].

Metabolism

Metabolism involves how a drug undergoes changes and generates metabolites. Uloric is mainly metabolized by the enzyme CYP 2C9. Other enzymes include CYP 1A1/1A2/2C8 and UGT 1A1/1A8/1A9 [11]. Metabolic forms of Uloric include 67M-1, 67M-2, and febuxostat acyl glucuronide [10]. The metabolism of Colcrys depends on P-glycoprotein transport and CYP enzyme [7]. Colcrys is primarily metabolized in the enteric and hepatic systems by cytochrome P450 3A4 (CYP450 3A4) [8]. Colcrys is advised not to be taken with grapefruit juice because grapefruit juice is an inhibitor of CYP450 3A4. Regarding the metabolism of Krystexxa, one study found that co-administration of Krystexxa with methotrexate increased the exposure of Krystexxa due to decreased levels of anti-pegloticase antibodies [10]. Tissue proteases metabolize Krystexxa into amino acids.

Elimination

Elimination involves how a drug is expelled from the body. Uloric is primarily eliminated via urine in the form of febuxostat acyl glucuronide. It is estimated that 25% of Uloric is excreted via feces [11]. The approximate half-life of Uloric is eight hours [5]. The primary elimination sites for the drug metabolites generated by Colcrys include the biliary system, intestine, and kidneys. Factors that can affect the elimination of Colcrys include hepatobiliary dysfunction and aging [8]. The half-life of Colcrys is around 20-40 hours [12]. The half-life of Krystexxa is estimated to be about two weeks [13]. The renal and hepatic impairments by Krystexxa have yet to be studied [10].

Pharmacodynamics of gout drugs: Uloric, Colcrys, and Krystexxa

Drugs that improve renal excretion of uric acid (uricosuric) or lower uric acid production (uricostatic) are primarily used for treating chronic gout, while either colchicine, corticosteroids, or NSAIDs are recommended options for treating acute gout attacks [14]. Depleting uric acid levels during an initial acute gout attack has been associated with a tendency to induce gout flares [15]. These contradictory flares are suspected to occur due to the activation of the alternative and classic complement pathways upon interaction with dissolved MSU crystals, which become unstable following the depletion of serum uric acid [16]. At concentrations above 7 mg/dL, serum uric acid becomes saturated, leading to the precipitation of MSU crystals within tissues and joints. The main goal of chronic gout treatment is to prevent MSU crystal precipitation by reducing serum uric acid levels to less than 6 mg/dL in individuals who have suffered from frequent gout attacks and damage due to high uric acid levels. Uricostatic drugs include xanthine oxidase inhibitors (such as Febuxostat), and recombinant uricases (such as pegloticase). Anti-inflammatory drugs such as colchicine are used in the prophylaxis of acute gout attacks. The direct effects that Uloric, Colcrys, and Krystexxa have on the body are highlighted below.

Uloric (Febuxostat)

Uloric was approved by the FDA in 2009 for managing hyperuricemia in patients with chronic gout. It functions by selectively inhibiting xanthine oxidase, which is involved in the catabolism of purines and specifically catalyzes the conversions of hypoxanthine to xanthine, and xanthine to plasma uric acid. Uloric binds to and occupies the molybdenum-pterin active site of xanthine oxidase [17] and thereby prevents substrate binding via mixed-type inhibition of both the oxidized and reduced xanthine oxidase isotypes, with inhibitor constant Ki and Ki' values of 0.6 and 3.1 nM respectively [18]. Allopurinol was previously the main xanthine oxidase inhibitor approved by the FDA but is known to produce adverse effects ranging from mild to severe (in which case it is known as allopurinol hypersensitivity syndrome). In contrast, Uloric is a non-purine xanthine oxidase inhibitor that has a negligible effect on the other enzymes involved in the purine and pyrimidine metabolism pathways [19]. In a study involving healthy subjects and subjects with mild or moderate hepatic impairment, it was found that both groups displayed substantial decreases in serum uric acid concentrations and renal clearance of xanthine and hypoxanthine over seven days following administration of a once-daily 80 mg dose of Uloric [20]. For adults, Uloric is currently recommended to be administered at doses of 40 mg daily and can be increased to 80 mg daily if serum urate levels do not reach an endpoint of less than 6 mg/dL within two weeks [6].

Colcrys (Colchicine)

Colcrys is an anti-inflammatory medication that was approved by the FDA in 1961 for acute gout flares and gout prophylaxis. It produces several biological effects that interfere with inflammation: inhibition of the activation of the nucleotide-binding domain, leucine-rich repeat-containing family pyrin domain containing 3 (NLRP3) in response to MSU crystals [21] and thereby inhibiting innate immune responses, binding tubulin and preventing microtubule polymerization, which in turn prevents the recruitment of neutrophils [22], and impairing mast cell degranulation [23] among others. Despite being used to treat acute gout for decades, there is an ill-defined distinction between toxic and therapeutic doses of colchicine. Colchicine has a narrow therapeutic index, and patients may experience gastrointestinal side effects before the intended clinical effect [24]. For prophylaxis of gout flares in adult patients, it is currently recommended that Colcrys be orally administered at 0.6 mg once or twice daily. For treating acute gout flares, 1.2 mg of Colcrys is to be taken orally during the initial attack, and an additional 0.6 mg is to be taken one hour following this administration [8].

Krystexxa (Pegloticase)

Krystexxa was approved by the FDA in 2010 for treating chronic gout in adult patients unresponsive or contraindicated to conventional gout therapies. It is a PEGylated uricase that catalyzes the conversion of uric acid to allantoin, thereby reducing uric acid concentration and preventing MSU crystal precipitation [25]. It has been found that two-hour, twice-a-week IV infusions of 8 mg Krystexxa led to subjects maintaining plasma uric acid levels of less than 6 mg/dL within three to six months of beginning this treatment, along with improvements in the frequency of gout flares and resolution of tophi [26]. Further studies are needed to elucidate the Tmax and half-life of Krystexxa. It is currently recommended for adult patients to provide 8 mg/mL once every two weeks as an IV infusion [9].

The targeted population of Uloric, Colcrys, and Krystexxa

Gout is classically associated with Caucasian, middle-aged obese males who follow a poor diet. However, this depiction misses many patients living with gout, particularly non-White populations, and women who may experience a higher incidence or prevalence of gout, more severe disease, and/or mismanagement [27]. The pathophysiology of gout is complex and has led to the development of drugs targeting multiple mechanisms of gout pathogenesis. This naturally means that the target population for individual gout drugs has narrowed down to best address patients' unique pathophysiology.

Uloric (Febuxostat)

Uloric is indicated for patients who have symptomatic chronic hyperuricemia with gout. It is not recommended for patients who have asymptomatic hyperuricemia. Special consideration for dosage must be made for patients with severe renal impairment. In this population, it is recommended to limit the dose to 40 mg once per day. Dose adjustment should not be considered in patients with mild or moderate renal or hepatic impairment [6]. Data on the use of Uloric during pregnancy is limited and it is currently not possible to establish the risk of adverse developmental outcomes. Additionally, no data has been collected regarding the accumulation of Uloric in breast milk, its effects on milk production, or the effect of the milk on infants [6]. In geriatric populations, dosage does not need to be adjusted, but studies still need to be conducted to establish safety and efficacy in pediatric populations and patients with secondary hyperuricemia. It is important to note that the results of the Cardiovascular Safety of Febuxostat and Allopurinol in Patients with Gout and Cardiovascular Morbidities (CARES) trial show that patients with gout and a pre-existing major cardiovascular disease have a higher risk of cardiovascular and all-cause mortality when compared to patients taking allopurinol [28].

Colcrys (Colchicine)

Colcrys is indicated for both the prophylaxis and treatment of gout flares. Because Colcrys has been approved for use in the United States for over 60 years, many long-term studies on the effect of and contraindications to prescribing Colcrys in different populations have been conducted. Use of Colcrys has not been associated with any major birth defects, miscarriage, or other bad outcomes related to pregnancy although most of this data is based on non-randomized studies without control arms [8]. Some epidemiology-based studies and case reports have associated colchicine use with infertility in males [8]. Colchicine has been found in human breast milk; however, there is no evidence that it causes harm in children who are breastfed [8]. Use in pediatric patients has not been associated with any poor outcomes, but there is a lack of studies looking into its efficacy and safety [8]. Use in geriatric populations over the age of 65 has not been studied well. These patients should be approached cautiously as many elderly patients also suffer from renal impairment, which has been shown to decrease the clearance of colchicine by 75% [8]. Patients who suffer from hepatic impairment may also be at risk of decreased colchicine clearance which must be considered. However, dose adjustment may not be necessary for patients with only mild to moderate hepatic impairment [8].

Krystexxa (Pegloticase)

Krystexxa is indicated only for adults with chronic gout refractory to conventional gout therapies such as xanthine oxidase inhibitors. These patients were either unable to have their uric acid levels normalized or symptoms controlled with maximum dosages of xanthine oxidase inhibitors [9]. There is currently no recommendation for dose adjustment in patients with renal impairment or in patients over the age of 65. No strong connections have been established between taking Krystexxa and birth defects in pregnancy. It is not currently known if the drug is excreted in breast milk, so it is not advised to breastfeed while using the drug unless a benefit can be established [9]. The safety of Krystexxa in pediatric populations has not been studied [9]. Patients at an increased risk of having glucose-6-dehydrogenase (G6PD), such as African or Mediterranean patients, should be screened before being prescribed Krystexxa because of the increased risk of developing methemoglobinemia or hemolysis [29].

Benefits and contraindications of Uloric, Colcrys, and Krystexxa

Uloric, Colcrys, and Krystexxa share some similarities in the treatment of gout. However, each of these drugs holds key differences that a healthcare provider should understand to deliver optimal treatment. Below are some primary benefits and contraindications of the indicated gout treatments.

Benefits

Uloric (febuxostat): Since approval for gout prevention and management, Uloric has displayed efficacy in lowering serum uric acid levels comparable to allopurinol, which has long been the standard drug in this regard [30]. Uloric can be safely used with other medications such as Colcrys and specific NSAIDs such as indomethacin to prevent initial acute gout flares that occur during the start of treatment with Uloric [31]. It can also be used without any consideration towards food or antacid usage and does not have to be dose-regulated for patients with mild or moderate renal impairments [32]. Based on the results of the Febuxostat versus Allopurinol Streamlined Trial (FAST) trial, the use of Uloric has not been shown to be associated with an increased risk of death or adverse events compared to allopurinol [33].

Colcrys (colchicine): The main benefits of using Colcrys come from its wide range of uses. While Colcrys is FDA-approved for gout, it treats familial Mediterranean fever effectively [6]. A variety of off-label uses of Colcrys include Paget's disease of bone, hepatic cirrhosis, primary biliary cirrhosis, acute/recurrent pericarditis, prevention of post pericardial syndrome, dermatitis herpetiformis, idiopathic pulmonary fibrosis, chronic immune thrombocytopenia, idiopathic thrombocytopenic purpura, and pseudogout [6]. This makes Colcrys a considerable drug choice when unable to obtain a synovial fluid analysis to distinguish gout from pseudogout. Unlike some other gout treatments, Colcrys is widely used for the treatment of acute gouty flares as well as for gout prophylaxis. Lastly, colchicine is available as an oral tablet and a topical gel [6].

Krystexxa (pegloticase): Krytexxa is an alternative option for patients who continue to experience chronic gouty arthritis with tophi and high serum uric acid levels despite treatment with a conventional urate-lowering therapy like Uloric [34]. The primary benefit of Krystexxa is that it lowers uric acid levels by converting uric acid into allantoin, a soluble metabolite readily excreted by the kidneys [12,35]. As a pegylated uricase, Krystexxa retains a longer half-life and requires less frequent administration than non-pegylated uricases [36]. Additionally, maximized solubility and reduced immunogenicity are also beneficial properties Krystexxa has as a result of being pegylated [12,35].

Precautions and Contraindications

Uloric (febuxostat): It has been demonstrated that xanthine oxidase inhibitors, including allopurinol metabolize thiopurine immunosuppressants, such as azathioprine (brand names: Azasan, Imuran), and mercaptopurine (brand name: Purinethol), leading to cytotoxicity and dose-related myelosuppression [37]. FDA labeling indicates that Uloric should not be taken in combination with either of these drugs. The CARES trial showed that there were higher rates of adverse cardiovascular events, including cardiovascular death, nonfatal myocardial infarction, nonfatal stroke, or urgent revascularization for unstable angina, in the febuxostat group compared to the allopurinol group [28]. Other reported adverse effects of Uloric include nausea, diarrhea, liver function abnormalities, and headaches [32].

Colcrys (colchicine): The contraindications of colchicine result from its dependence on being metabolized by P-glycoprotein transport and CYP 384 isoenzymes. Furthermore, P-glycoprotein and CYP3A4 inhibitors can dramatically increase colchicine in the plasma [6]. The metabolism of colchicine occurs primarily in the liver. The liver then eliminates colchicine through biliary pathways, and the final elimination occurs via the urine. Therefore, decreased renal and/or hepatic function with P-glycoprotein or CYP3A4 inhibitors are contraindicated due to their ability to increase concentrations of colchicine in the plasma [6]. Increased serum creatine, bone marrow suppression, myelosuppression, proximal weakness (myeloneuropathy), and rhabdomyolysis can all result from increased plasma colchicine.

Colchicine should be used cautiously for alcoholic, pregnant, pediatric, and geriatric populations. Patients on dialysis should have a dosage reduction due to the inability to remove colchicine via dialysis. Lastly, related to associated myelosuppression, patients with dental disease should avoid colchicine as a treatment option for gout [6].

Krystexxa (pegloticase): The updated contraindications for Krystexxa that the FDA has reported include patients with G6PD deficiency and those with a history of a serious hypersensitivity reaction [9]. Precautions associated with Krystexxa include infusion reactions, anaphylaxis, G6PD-associated hemolysis and methemoglobinemia, gout flares, and congestive heart failure [9]. G6PD deficiency is contraindicated with Krystexxa because of a by-product that is generated when Krystexxa converts uric acid into allantoin, which is hydrogen peroxide [35]. G6PD protects typically against the effects of oxidative stress by providing nicotinamide adenine dinucleotide phosphate hydrogen (NADPH) for the oxidation of glutathione, which generates the substrate for glutathione peroxidase [36]. Glutathione peroxidase catalyzes the oxidation of hydrogen peroxide into water. Erythrocytes primarily rely on G6PD for NADPH, and low levels of NADPH resulting from G6PD deficiency can cause hemolytic anemia [35,34]. Methemoglobinemia is another condition resulting from G6PD deficiency because NADPH is required by the enzyme that reduces methemoglobin [38]. For patients with a history of serious hypersensitivity, antihistamines, and corticosteroids are administered before treatment with Krystexxa [35,34].

Table 1 shows a summary of Uloric, Colcrys, and Krystexxa, allowing for a side-by-side comparison of each medication class, route of administration, primary usage, and primary contraindications. The drug class is based on each medication's primary mechanism of action. While there are several ways to administer each drug, the table focuses on the administration most frequently used in practice. Primary usage refers to what classification of gout the given drug is primarily used to treat. Lastly, the primary contraindications section of the table illustrates some of the most common contraindications for each given drug.

**Table 1:** Comparison of Uloric, Colchicine, and Krystexxa in gout treatment References: [6,9,10]

	Drug class	Route of administration	Primary usage	Primary contraindications
Uloric (febuxostat)	Xanthine Oxidase Inhibitor	Oral tablet	Chronic gout treatment	Hepatic failure, Cardiovascular thromboembolic events, Use of azathioprine or mercaptopurine
Colcrys (colchicine)	Anti-inflammatory	Oral tablet	Acute gout treatment and prophylaxis	Renal disease, Hepatic disease, Hepatic biliary obstruction
Krystexxa (pegloticase)	Enzyme	IV infusion	Chronic gout treatment	Glucose-6-phosphate dehydrogenase (G6PD) deficiency, History of serious hypersensitivity reactions

## Conclusions

Gout hinders the quality of life for thousands worldwide each day, and different means of treatment can provide an improved standard of living for those affected. Uloric, a recently approved non-purine xanthine oxidase inhibitor, is an efficacious alternative for preventing gout in patients who cannot tolerate allopurinol. Colchicine is a well-documented historical treatment for gout, and Colcrys is an oral colchicine medication approved for treating and prophylaxis of acute gout. Krystexxa is a recently approved IV medication for patients unresponsive to standard first and second-line treatments for chronic gout. Due to Krystexxa's relatively short time as a gout treatment, additional studies are needed to understand its pharmacokinetic properties better. Each of these three drugs has unique mechanisms of action compared to each other and is currently in use in the United States. The three gout drugs, Uloric, Colcrys, and Krystexxa, add to the variety of treatment options for gout, and understanding their differences allows for optimal treatment of gout patients.
